# Spoligotyping-based molecular typing of *Mycobacterium tuberculosis* complex isolated from Metahara sugar factory workers in Central Ethiopia

**DOI:** 10.3389/fmed.2025.1641535

**Published:** 2025-09-01

**Authors:** Tilahun Bogale, Temesgen Mohammed, Aboma Zewude, Hazim O. Khalifa, Gobena Ameni

**Affiliations:** ^1^LeDeG Midwifery College Charity Organization, Addis Ababa, Ethiopia; ^2^Aklilu Lemma Institute of Pathobiology, Addis Ababa University, Addis Ababa, Ethiopia; ^3^Department of Veterinary Medicine, College of Agriculture and Veterinary Medicine, United Arab Emirate University, Al Ain, United Arab Emirates; ^4^Department of Pharmacology, Faculty of Veterinary Medicine, Kafrelsheikh University, Kafr El-Sheikh, Egypt

**Keywords:** *Mycobacterium tuberculosis* complex, genetic diversity, spoligotyping, tuberculosis, Metahara, Ethiopia

## Abstract

**Background:**

Understanding the genetic makeup of *Mycobacterium tuberculosis* complex (MTBC) strains is crucial, as lineage differences influence transmissibility, pathogenicity, and drug resistance patterns, all of which are essential for understanding MTBC transmission dynamics and designing effective TB control strategies. The present study investigated the genetic diversity of *Mycobacterium tuberculosis* complex among pulmonary tuberculosis (TB) patients employed at Metahara Sugar Factory, located in Fentale district, East Showa Zone Oromia, central Ethiopia.

**Methods:**

A cross-sectional study was conducted among 390 suspected pulmonary TB patients. Sputum samples were examined using Ziehl-Neelsen staining and cultured, followed by molecular characterizations of the isolates using region of difference 9 (RD9) deletion typing and spoligotyping.

**Results:**

Out of 390 participants, 96 (24.6%) were smear positive, and 89 (22.8%) were culture positive. RD9 deletion typing confirmed 88 isolates as *M. tuberculosis*. Further characterization of the 88 isolates using spoligotyping revealed 28 distinct spoligotyping patterns of which 15 unique (single isolates), and 13 shared among 73 clustered isolates. Among these, 19 matched shared international type (SITs) in the SpolDB4 database, while, 9 were novel (orphan) patterns. The predominant SITs were SIT523 (19.32%), SIT53 (13.6%), SIT149 (9.1%) and SIT289 (7.95%). Lineage analysis using TB-insight RUN TB-Lineage classified the strains primarily as Euro-American (63.64%), followed by Indo-Oceanic (20.45%), East-African-Indian (14.77%) and *M. africanum* (1.14%).

**Conclusion:**

The high clustering rate observed may suggest recent transmission; however, this must be interpreted cautiously due to the limited discriminatory power of spoligotyping, which may overestimate clustering and underestimate diversity. This underscores the need for targeted TB control strategies informed by enhanced molecular surveillance.

## Introduction

Tuberculosis (TB) caused by *Mycobacterium tuberculosis* (Mtb) complex, is a major global public health challenge, with an estimated one-quarter of the world’s population infected and at risk of developing active disease during their lifetime (1). TB remains one of the leading causes of morbidity and mortality worldwide. Despite ongoing control efforts, it continues to exert significant health and socio-economic burdens (1, 2). According to the WHO Global TB Report 2024, approximately 10.8 million people developed TB globally. Although TB-related deaths have decreased compared to the previous 2 years, TB has once again become the leading cause of death from a single infectious agent (2). While the global incidence of TB has been gradually declining, the emergence and spread of multidrug-resistant TB (MDR-TB) complicate control strategies and treatment outcomes (1). In Ethiopia, tuberculosis continues to pose a major public health threat, particularly in settings with limited diagnostic and treatment capacity. Industrial settings such as Metahara may face TB control challenges due to factors like limited access to timely diagnosis and high worker turnover. However, evidence of overcrowding or the presence of internally displaced populations (IDPs) within the factory environment is limited, and therefore not assumed in this study. However, these barriers complicate national efforts to achieve the End TB Strategy goal of eliminating the disease by 2035 (3).

TB disproportionately affects low- and middle-income countries, especially in regions like sub-Saharan Africa and Southeast Asia, where TB burden is high due to factors such as the HIV/AIDS epidemic, poverty, overcrowding, malnutrition, and inadequate healthcare infrastructure (4, 5). In Ethiopia, tuberculosis (TB) continues to cause substantial morbidity and mortality. According to the WHO Global TB Report 2023, the country remains among the 30 highest burden countries for TB and TB/HIV co-infection (1). In 2022, the country reported an estimated 170,000 new TB cases, with an incidence rate of 148 per 100,000 population (1, 6). Although TB/HIV co-infection rates have declined due to integrated interventions, national data still indicate co-infection rates of around 7–8% (7). Contributing factors such as poverty, delayed healthcare-seeking behavior, overcrowded living conditions, and malnutrition continue to drive TB transmission and mortality (1).

Despite Ethiopia being a high TB burden country, data on the molecular characteristics of *M. tuberculosis* strains and lineages remain limited, especially in occupational settings. Industrial laborers, including sugar factory workers, are often exposed to environmental and social risk factors such as close living quarters, dust exposure, and population mobility that may facilitate TB transmission. Although several molecular epidemiological studies in Ethiopia have focused on the general population, pastoralist communities, and urban residents, limited data exist on tuberculosis (TB) strain diversity among high-risk occupational groups such as factory workers (8–10). Understanding the molecular characteristics of circulating MTBC strains in these settings is essential for elucidating transmission dynamics and designing effective, targeted control strategies. Moreover, identifying strain types in specific geographic and occupational contexts contributes to the global TB knowledge base and supports efforts to control and eliminate the disease. Therefore, the present study aimed to characterize the MTBC species and strains causing pulmonary tuberculosis among workers at the Metahara Sugar Factory, located in the Fentale District, East Shewa Zone, Oromia Regional State, central Ethiopia.

## Materials and methods

### Study area

**Figure 1:**
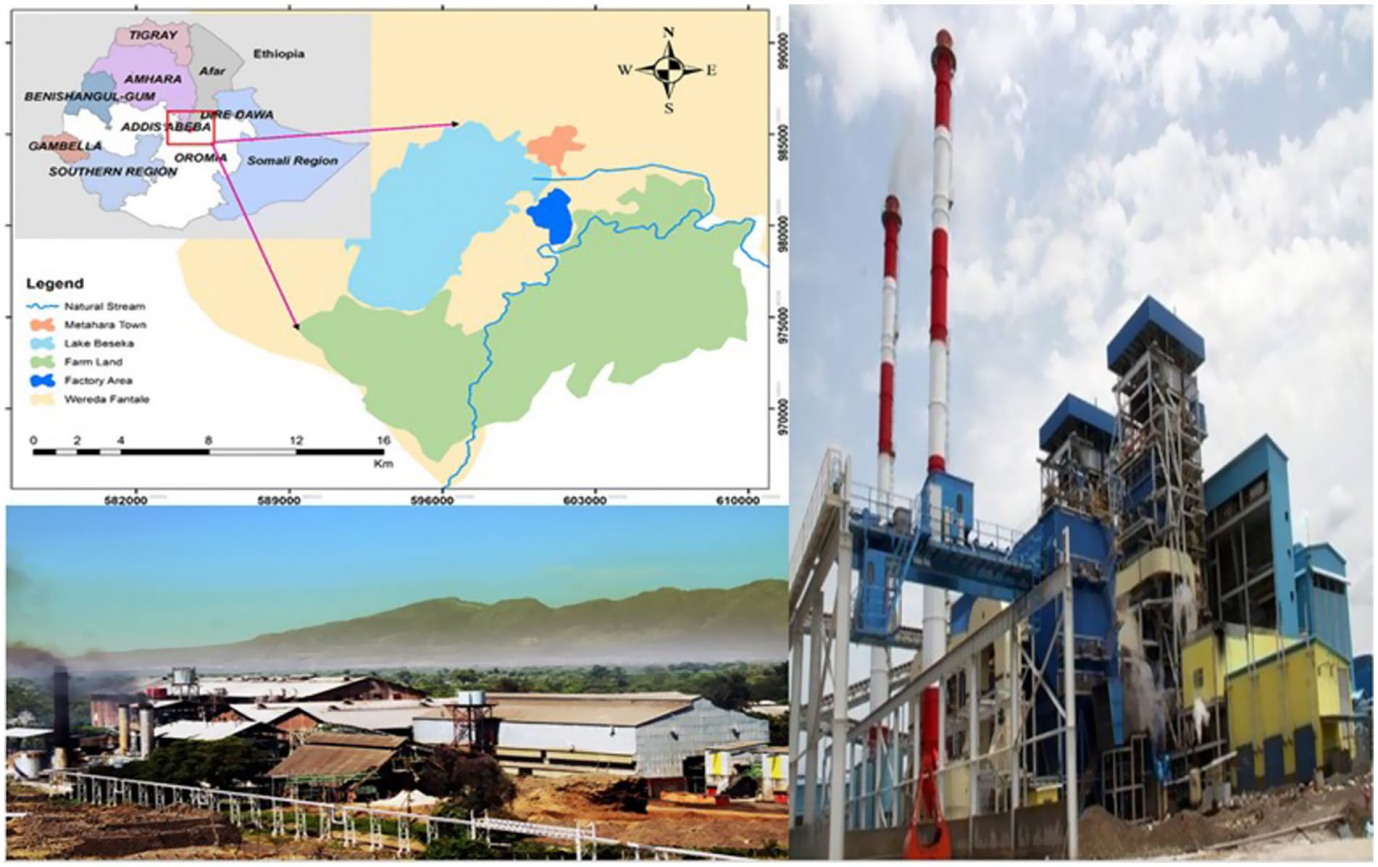
Map of the location of the Metehara Sugar Factory in central Ethiopia, including the sugarcane farm and factory. Reproduced from Fito et al. (40) with permission from the publisher.

The Study was conducted in Fentale district, East Showa Zone of Oromia Regional State, central Ethiopia, which is located at 96 and 200kms from Adama and Addis Ababa, respectively, on the main road from Addis Ababa to Djibouti. The district covers an area of 1,532 km^2^ and includes 18 rural kebeles, two towns, and the Metehara Sugar Factory residential compound, with a total population of approximately 81,740 (43,266 males and 38,474 females) based on the 2007 national census (11) (Figure 1). The climate is hot and arid, with daily temperatures ranging from 37°C to 40°C. Most of the area lies at an elevation of around 1,000 meters above sea level. Metehara Sugar Factory, located within the district, is one of the oldest and largest sugar production facilities in the country, with a large workforce and extensive residential quarters. Established in 1970, it operates over 10,000 hectares of sugarcane plantations and produces sugar, ethanol, and electricity. During the study period (2014–2016), the factory employed a large workforce comprising both permanent and seasonal workers. While exact historical employment records were not available, unofficial estimates suggest that several thousand employees worked at the facility, many of whom lived in shared accommodations within the factory compound conditions that may increase the risk of TB transmission. This setting provided a relevant occupational environment for investigating the molecular epidemiology of tuberculosis.

### Study design and period

A cross-sectional study was conducted from July 2014 to August 2016 (Gregorian calendar) to identify the spoligotype international types (SITs) of *Mycobacterium tuberculosis* complex (MTBC) isolates responsible for pulmonary TB (PTB) among factory workers.

### Study population

A total of 390 presumptive pulmonary TB cases were recruited over a two-year period from July 2014 to August 2016 using a non-probability convenience sampling approach. Participants were enrolled as they presented to the Metehara Sugar Factory clinic or Merti Hospital with symptoms suggestive of TB, particularly a cough lasting for 2 weeks or more, in line with national TB case screening guidelines. Additional inclusion criteria were age ≥15 years and residency or employment within the factory compound. The age threshold was applied because the study targeted adolescents and adults, consistent with national TB surveillance protocols, and while the majority of participants were factory workers, some were family members residing in the compound. Individuals previously treated for TB were excluded to minimize potential confounding from mixed infections, relapse, or treatment-induced strain selection, which could affect the accuracy of strain clustering and lineage analysis. Patients unable to provide sputum samples were also excluded. No mass screening was conducted inside the factory; all participants were self-presenting patients.

### Sample collection and mycobacterial isolation

Sputum samples were collected from each participant following the World Health Organization’s 2015 guidelines for TB surveillance (12). Each patient was instructed to provide two sputum specimens (one spot and one early morning) in sterile, leak-proof, screw-capped containers. Patients were advised to produce deep cough sputum, not saliva, in a well-ventilated area or designated sputum collection booth. Samples were labeled and initially examined for acid-fast bacilli (AFB) using Ziehl-Neelsen (ZN) staining at the point-of-care laboratories within the Metehara Sugar Factory clinic and Merti Hospital. Due to limited resources, only the remaining portions of ZN smear-positive samples were stored at −20°C at the collection site and later transported to the TB laboratory at Aklilu Lemma Institute of Pathobiology (ALIPB), Addis Ababa University, for culture and molecular characterization. Transportation was done in a cold chain using an icebox maintained at +4°C to preserve the integrity of the specimens for mycobacterial culture. We acknowledge that excluding smear-negative specimens from culture may have led to an underestimation of the culture positivity rate and genetic diversity, as smear microscopy is less sensitive than culture.

The sputum samples were processed (decontaminated and neutralized) for culturing according the standard operating procedure described earlier (13). Briefly, equal volume of 4% NaOH was mixed with sputum sample, and the mixture was centrifuged at 3000 rpm for 15 min at +4°C. After decanting the supernatant, the sediment was neutralized with 2 N HCl using phenol red as an indicator. Neutralization was achieved when the color of the solution was changed from purple to yellow. Thereafter, 100 μL of the suspension was inoculated onto two sterile LJ medium slopes (which were enriched with either pyruvate or glycerol). The inoculated media were then incubated at 37°C in slanted position for 1 week and upright position for four to 5 weeks. Growth of mycobacteria was monitored every week for up to 8 weeks. Slants with no evidence of growth after 8 weeks were considered negative (14). Specimens with growth of colonies were examined for the acid-fast bacilli after staining with Ziehl Neelsen stain. Colonies positive for acid-fast bacilli (AFB) were harvested and re-suspended in 200 μL sterile distilled water. Thereafter, the suspension was inactivated by heating at 80°C for 45 min in water bath for the release of DNA (9).

### Molecular typing

Molecular typing was performed on isolates obtained from smear-positive and culture-confirmed cases to ensure adequate bacillary load. Due to resource limitations, smear-negative but culture-positive cases were not included in molecular typing, which may have introduced a selection bias. This is noted as a limitation of the study. All molecular procedures were conducted under strict aseptic conditions using dedicated pre- and post-PCR work areas. Negative controls were included in each PCR run to monitor for contamination. To further minimize the risk of cross-contamination, we used sterile, filtered tips and separate pipettes for each stage of the molecular workflow.

### Region of difference (RD) 9-based polymerase chain reaction (PCR)

Identification of *M. tuberculosis* from the other members of *M. tuberculosis* complex species was done using RD9-based PCR. RD9-PCR was performed on heat-killed cells to confirm the presence or absence of RD9 using three primers namely, RD9flankF, RD9 IntR, and RD9flankR. Amplification was done by standard thermo cycler (VWR Thermo cycler, UK). The PCR amplification mixture used consisted of 10 μL HotStar Taq Master Mix (Qiagen, United Kingdom), 7.1 μL distilled water, 0.3 μL of each three primers and 2 μL of DNA template (heat killed cells), giving a total volume of 20 μL. The PCR reaction was heated at 95oC for 15 min after which it was subjected to 35 cycles consisting of 95°C for 1 min, 55°C for 1 min, and 72°C for 1 min. Thereafter, the reaction mixture was maintained at 72°C for 10 min following which the product was removed from the thermo cycler and detected by agarose gel electrophoresis. The PCR amplification product was run by agarose gel electrophoresis in 1.5% agarose gel in 1 × Tris Borate-EDTA (TBE) running buffer at 110 V and 400 mA for 35 min. Ethidium bromide at a ratio of 1:10, 100 base pair (bp) DNA ladder and orange 6 × loading dye were used in gel electrophoresis and the gel was visualized. The gel was then visualized using a computerized Multi- Image Light Cabinet (VWR). *M. tuberculosis* H37Rv, *M. bovis* bacille Calmette-Guérin, and water were included as positive and negative controls. Interpretation of the result was based on bands of different sizes, as previously described by (15).

### Spoligotyping of mycobacterial isolates

Spoligotyping was performed on 88 *M. tuberculosis* complex isolates at the Aklilu Lemma Institute of Pathobiology, Addis Ababa University, following the standard operating procedure that was used by Berg et al. (16) and primarily developed by Kamerbeek et al. (17). The DNA released by heat-killing of the colonies was used as a template to amplify the direct repeat (DR) region of *M. tuberculosis* complex by polymerase chain reaction (PCR) using oligonucleotide primers derived from the DR sequence, RDa (5’GGTTTTGGGTTTGAACGAC3’) and RDb (5’CCGAGAGGGGACG GAAAC3’) primers (17). The total volume of the reaction the PCR reaction mixture was 25 μL and constituted of 12.5 μL of HotStarTaq Master Mix (Qiagen; this solution provides a final concentration of 1.5 mM MgCl2 and 200 mM of each deoxyribonucleotide triphosphate), 2 μL of each primer (20 pmol each), 5 μL suspension of DNA template (approximately 10–50 ng), and 3.5 μL Qiagen water. The mixture was heated for 15 min at 96°C and then subjected to 30 cycles of 1 min at 96°C, 1 min at 55°C, and 30 s at 72°C, and the final extension at 72°C for 10 min.

Immediately before running the spoligotyping, the PCR product was denatured using thermocyler at 96°C for 10 min and then removed from the thermocycler and kept on ice so as to prevent denaturing of the PCR products. Thereafter, the denatured PCR product was loaded onto a membrane covalently bounded with a set of 43 oligonucleotides, each corresponding to one of the unique spacer DNA sequences within the DR locus of *M. tuberculosis* complex and then hybridized at 60^o^c for 1 h. After hybridization, the membrane was washed twice for 10 min in 2x SSPE (1x SSPE is 0.18 M NaCl, 10 mM NaH_2_PO_4_, and 1 mM EDTA [pH 7.7])-0.5% sodium dodecyl sulfate at 60^o^C and then incubated in 1:4000 diluted streptavidin peroxidase (Boehringer) for 1 h at 42°C. The membrane was washed twice for 10 min in 2 x SSPE-0.5% sodium dodecyl sulfate at 42°C and rinsed with 2 x SSPE for 5 min at room temperature. Hybridizing DNA was detected by the enhanced chemiluminescence method (Amersham, Biosciences, Amersham, UK) and by exposure to X-ray film (Hyperfilm ECL, Amersham). A mixture of 10 mL of ECL reagent 1 and 10 mL of ECL reagent 2 was prepared, and then added onto the membrane, and the membrane was rinsed in the solution for 5 min at room temperature. Then, the membrane was attached onto a film in the dark room and placed in the cassette and incubated for 15 min at room temperature. Thereafter the film was removed and placed in a developer solution for 2 min. The film was removed from the developer and rinsed with tap water for 15 s and then placed in a fixer solution for 1 min. Thereafter, the film was dried and used for interpretation of the result. The presence of the spacer was identified as a black square while the absence of the spacer was identified as a white square on the film. Thereafter, the black squares were converted to 1 while the white squares were converted to 0 and then transferred to the spoligotype international types-VNTR international types (SITVIT) database for the identification of the spoligotype international types (SIT) and the lineages of the isolates.

### Data management and analysis

All data generated during laboratory investigations were double-entered and cleaned using Microsoft Excel 2016. Descriptive statistical analysis was performed using SPSS version 25. The results of spoligotyping were converted into octal and binary formats, and then entered into query box so that the names of the strains are retrieved from the database if the spoligotype pattern of the strain in question fits the pattern that has already been registered in the SPolDB4 database and at https://www.pasteur-guadeloupe.fr/SITVIT2/ [SITVIT1Database (14)]. If the pattern of the strain in question has not been registered in the database prior to this study, the strain was considered as an orphan. The lineages were also generated by entering binary and octal formats into the query box of SITVIT1Database and Run TB-Lineage.

## Results

### Sociodemographic characteristics

A total of 390 presumptive pulmonary TB cases were enrolled in the study, all of whom were either employees of the Metehara Sugar Factory or residents living within the factory’s residential compound. Recruitment was conducted over a two-year period (July 2014 to August 2016) through the factory’s on-site clinic and Merti Hospital both of which primarily serve the factory’s workforce and their families. The study team did not conduct active TB screening within the factory premises. Instead, participants were identified passively as they presented to these facilities for routine medical care with TB-suggestive symptoms. Individuals were considered presumptive TB cases based on having a persistent cough lasting more than 2 weeks, in accordance with national TB screening guidelines. Only individuals aged 18 years or older who were verified as factory employees or residents of the factory compound were included. Suspected TB patients from surrounding rural areas or referred from other facilities were excluded to maintain a focused occupational cohort.

Of the 390 study participants, 227 (58.2%) were male and 163 (41.8%) were female. The most represented age groups were 25–34 years (141 participants, 36.2%) and 35–44 years (139 participants, 35.7%). Among the 390 presumptive pulmonary TB cases enrolled, 96 individuals (24.6%) were smear-positive based on Ziehl-Neelsen staining. Of these, 89 (92.7%) were confirmed as culture-positive for *Mycobacterium tuberculosis* complex, representing a TB confirmation rate of 22.8% among the total study population. Due to resource constraints, smear-negative samples were not cultured or further analyzed, and clinical or radiological diagnosis alone was not used to define confirmed TB in this study (Tables 1, 2). Of the 96 smear-positive cases, 89 (92.7%) were culture-positive. The remaining 7 samples that failed to grow in culture may have been affected by factors such as over-decontamination, poor sample quality, or loss of viability during storage and transport. Additionally, smear-negative individuals were not cultured, which limited the detection of additional culture-positive cases and likely led to underreporting of TB prevalence in the study population. No cultured samples were excluded due to contamination. All 89 culture-positive isolates were confirmed to be members of the *Mycobacterium tuberculosis* complex (MTBC) based on RD9 deletion typing. No non-tuberculous mycobacteria (NTM) or other bacterial species were isolated.

**Table 1:** Distribution of culture-positive TB cases among the study participants by sex and age.

Characteristics	Total number of study participants	Number of culture positive	Percentage (%)
Sex			
Male	227	52	22.9
Female	163	37	22.7
Age year			
15–24	30	3	10.0
25–34	141	37	26.24
35–44	139	31	22.3
>45	80	18	22.5

**Table 2:** Summary of samples processed at each diagnostic and molecular step.

Sample type	Number of samples	Percentage (%)
Total TB suspects screened	390	100
Smear-positive (ZN staining)	96	24.6
Culture-positive	89	92.7 of Smear-positive
RD9 PCR-positive (*M. tuberculosis*)	88	98.9 of Culture-positive
Spoligotyping successfully typed	88	100.0 of PCR-positive

### Region of difference (RD) 9-based polymerase chain reaction (PCR)

**Figure 2:**
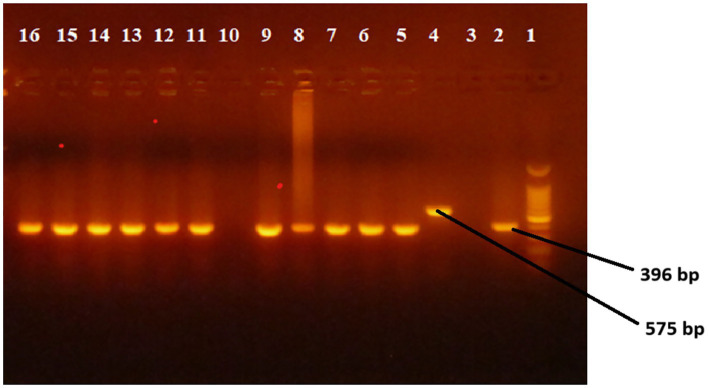
Picture of gel electrophoresis for RD9 deletion typing of *M. tuberculosis* isolates. Lane 1 is a ladder, lane 2 is *M. tuberculosis* control, lane 3 is negative control (molecular grade H2O), lane 4 is *M. bovis* control and lane 5–16 is culture isolate of *M. tuberculosis*,

Molecular characterization of the isolates using RD9 deletion typing showed that 88 of the isolates had intact RD9 implying that all the isolates were *M. tuberculosis* (Table 2 and Figure 2).

### Spolygotyping

**Figure 3:**
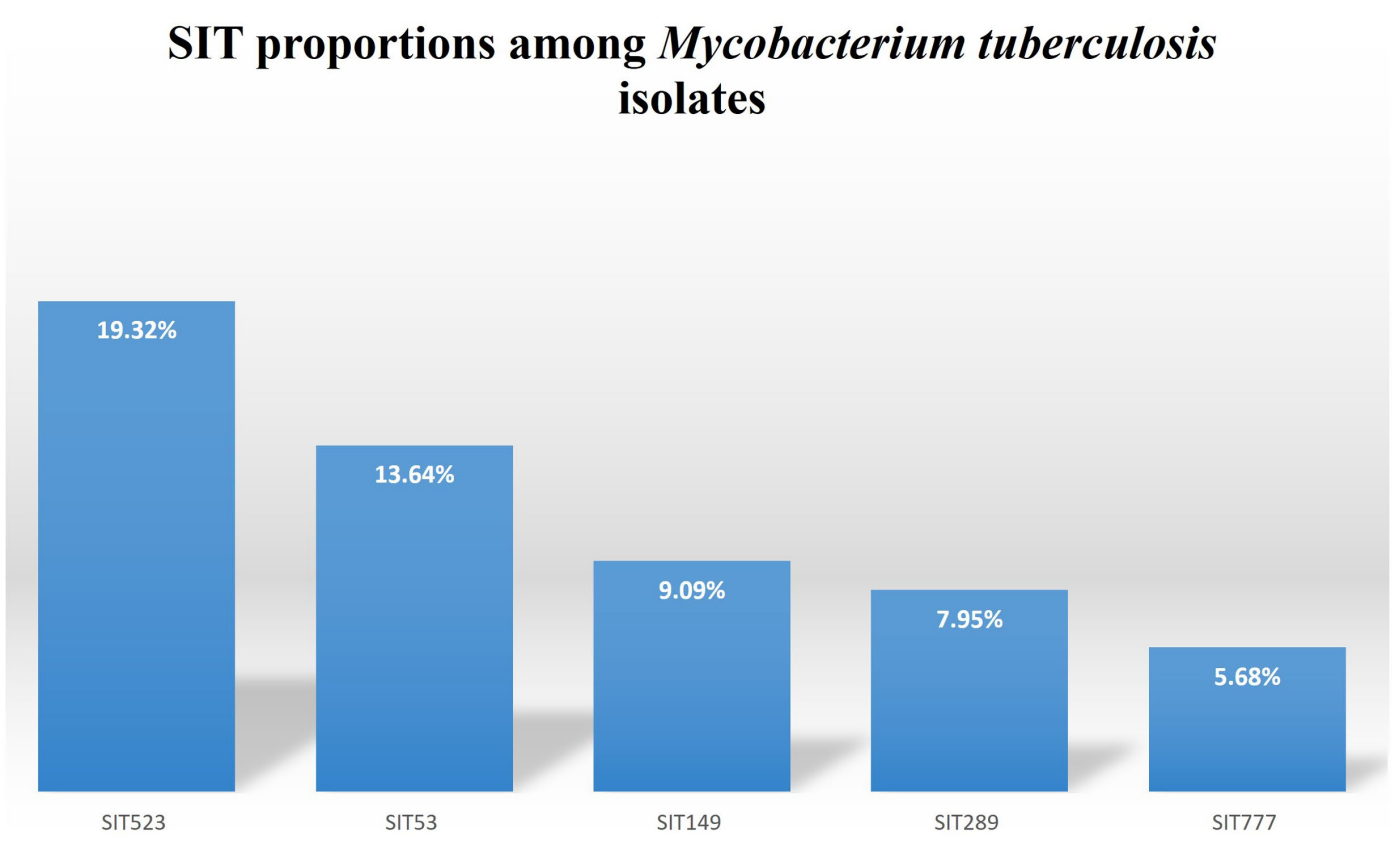
Bar chart showing the distribution of the top 5 Spoligotype International Types (SITs) identified among *Mycobacterium tuberculosis* isolates from sugar factory workers in central Ethiopia.

**Figure 4:**
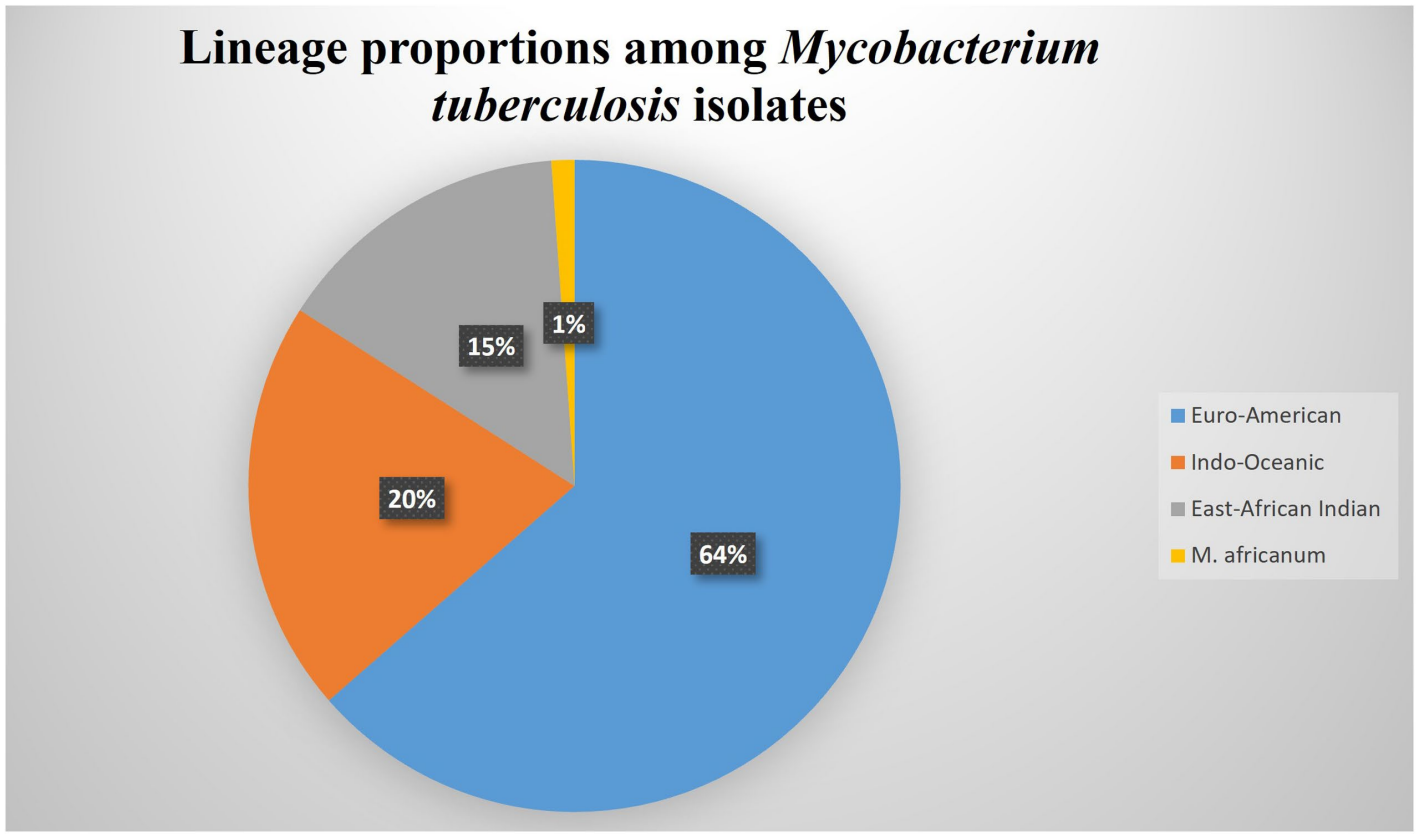
Pie chart showing the proportional distribution of *Mycobacterium tuberculosis* lineages identified among isolates from sugar factory workers in central Ethiopia.

Spoligotyping of the 88 isolates yielded 28 different spolygotype patterns. Of the 88 *M. tuberculosis* isolates analyzed, 73 (82.9%) were grouped into 13 clustered spoligotype patterns, while the remaining 15 isolates (17.1%) had unique patterns (Table 3 and Table 4). Based on this, the recent transmission Index (RTI) was calculated as 0.682, indicating that approximately 68% of TB cases may be attributable to recent transmission events. The proportion of unique spoligotype patterns among total isolates was 31.8%, indicating limited genetic diversity. Out of the 28 spoligotype patterns (strains), 19 strains associated with 75 isolates matched the preexisting patterns in the SITVIT2 database while the remaining nine spoligotype patterns associated with 13 isolates were not registered in the international spoligotype SITVIT2 database and thus designated as orphan strains (Table 4). The dominantly identified strains were SIT523, SIT53, SIT149 and SIT289, consisting of 17 (19.32%), 12 (13.6%), 8 (9.1%), and 7 (7.95%) isolates, respectively (Table 3 and Figure 3). Classification of the spoligotype patterns using TB-insight RUN TB-Lineage revealed that 63.64% of the isolates were Euro-American lineage followed by Indo Oceanic, East-African Indian (CAS) and *M. africanum* with 20.45, 14.77 and 1.14%, respectively, (Table 5 and Figure 4).

**Table 3:** Description of 19 shared types (SITs; *n* = 75 isolates) which have already been registered in the SITVIT2 or SpolDB4 database and corresponding spoligotyping defined lineages/sublineages starting from a total of 88 *M. tuberculosis* strains isolated in Fentale District East Showa Zone.

No	SIT	Isolates with similar pattern	CBN* Lineage	SITVIT2 Lineage/sublineage	Octal number	Binary format
1	523	17	IO	Manu1	777777777777771	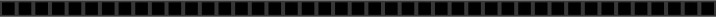
2	53	12	EA	Manu2	777777777760771	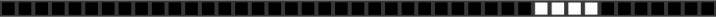
3	149	8	EA	T3-ETH	77700377760771	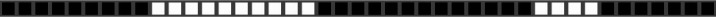
4	289	7	EAI	CAS1-Delhi	703777740003571	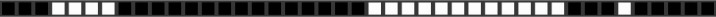
5	777	5	EA	H3-Ural-1	777777777420771	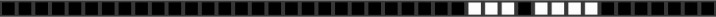
6	37	5	EA	T3	777737777760771	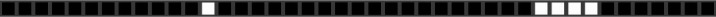
7	54	4	EA	Manu2	777777777763771	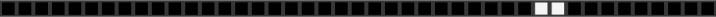
8	336	4	EA	X1	777776777760731	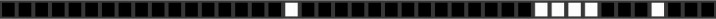
9	121	2	EA	H3	777777775720771	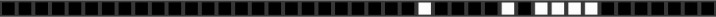
10	21	2	EAI	CAS1-Kili	703377400001771	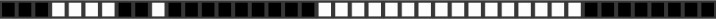
11	7	1	EA	T1	377777777760771	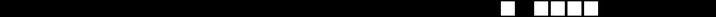
12	50	1	EA	H3	777777777720771	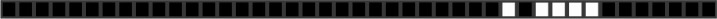
13	357	1	EAI	CAS1-Delhi	703777740000771	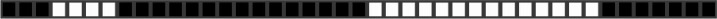
14	127	1	EA	H4-Ural-2	577777777420771	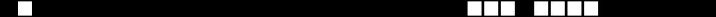
15	504	1	EAI	CAS1-Delhi	703777740003771	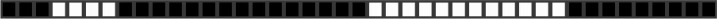
16	1821	1	EA	T	777347777760771	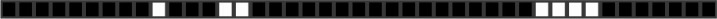
17	462	1	EA	T	777777777560771	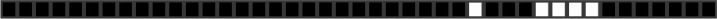
18	221	1	EA	X1	777766777760771	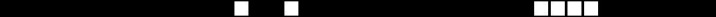
19	1,634	1	EA	Manu2	777777777723771	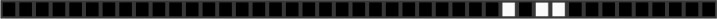

EA, Euro-American; EAI, East-African Indian; IO, Indo-Oceanic; MA, *M. africanum*; MB, *M. bovis*; CBN, conformal Bayesian network; KBBN, knowledge-based Bayesian network.

**Table 4:** Description of 9 orphan strains (*N* = 13) and corresponding spoligotyping defined lineages/sublineages recorded among *M. tuberculosis* strains starting from a total of 88 *M. tuberculosis* isolated in Fentale District East Showa Zone.

No	SIT	Isolates with similar pattern	CBN* Lineage	SITVIT2 Lineage/sublineage	Octal number	Binary format
1	Orphan	1	*M. africanum*	AFRI_1	773767577776670	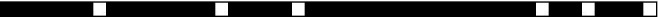
2	Orphan	1	EA	H3	777773775720771	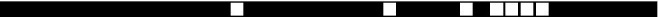
3	Orphan	2	EAI	CAS1-Delhi	703737740000771	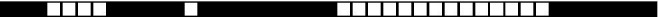
4	Orphan	1	EA	T	555347777740361	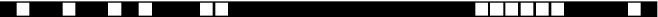
5	Orphan	2	EA	Manu2	777777777763571	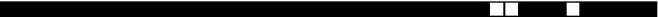
6	Orphan	1	EA	T1-RUS2	760000001760771	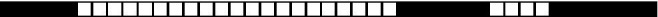
7	Orphan	3	EA	X1	777776777740731	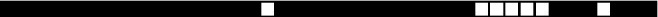
8	Orphan	1	IO	Manu1	577747777777761	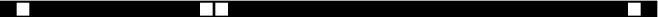
9	Orphan	1	EA	H	740000000360771	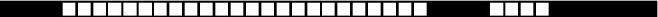

EA, Euro-American; EAI, East-African Indian; IO, Indo-Oceanic; MA, *M. africanum*; MB, *M. bovis*; CBN, conformal Bayesian network; KBBN, knowledge-based Bayesian network.

**Table 5:** Distribution of *M. tuberculosis* isolates by major lineage (*n* = 88).

Lineage	Number of isolates	Percentage (%)
Euro-American	56	63.64
Indo-Oceanic	18	20.45
East-African Indian	13	14.77
*M. africanum*	1	1.14
Total	88	100.0

## Discussion

This study revealed a high clustering rate (82.95%), suggesting active ongoing transmission of tuberculosis (TB) among workers at the Metahara Sugar Factory in central Ethiopia. Spoligotyping identified 28 distinct spoligotype patterns, dominated by strains SIT523, SIT53, SIT149, and SIT289. The isolates primarily belonged to the Euro-American lineage (63.64%), with Indo-Oceanic, East-African-Indian (CAS), and *M. africanum* lineages also identified.

Spoligotyping of 88 mycobacterial isolates revealed 28 distinct spolygotype patterns, which corresponded to 31.8% of genotype diversity. The diversity of spoligotypes strains that we observed in the present study was consistent with the study reported by (18–20) but lower than the percentages reported earlier by other studies in Ethiopia (26). The relatively low spoligotype diversity observed suggests that a limited number of *M. tuberculosis* lineages dominate transmission within the Metahara Sugar Factory community. Although migration can contribute to the introduction of diverse MTBC strains, the limited genetic diversity observed in this study may suggest ongoing local transmission dominated by a few well-adapted strains. While the Metehara Sugar Factory employs both permanent and seasonal workers, we did not collect specific data on employee turnover or migration history. Therefore, any interpretation regarding the role of transient labor migration in shaping strain diversity remains speculative and should be interpreted with caution.

Seventy-three of the mycobacterial isolates were grouped into 13 clusters with an overall clustering percentage of 82.95%. The clustering rate observed in this study was comparable with previous studies from Ethiopia (25, 27–30). However, it was higher than those reported previously by several other national studies (18, 21–24, 31–33). The difference in clustering rate among different studies could be due to the differences in population density of the study area, socioeconomic status of the study subjects, and the mobility of the population of the study areas (34). High level of strain clustering could suggest recent and ongoing TB transmission in the study area (35–37). A recent TB transmission index in a study population can be used to assess the efficacy of the TB control program in a certain geographical area (35, 38). The significant clustering in this study suggests ongoing TB transmission in the study area, which could be reflective of a recent outbreak or high rates of close contact among individuals. The clustering index is often used as an epidemiological marker to evaluate the effectiveness of TB control programs. In this context, it is concerning that despite the successful implementation of Ethiopia’s health extension program, which facilitates early detection and referral of TB cases for diagnosis and treatment, the clustering rate remains high, suggesting persistent transmission. The high clustering rate in the current study could be linked to the increasing incidence and prevalence of TB in Ethiopia, as well as to the social and economic factors that facilitate TB spread.

In the present study the dominantly identified strains were SIT523, SIT53, SIT149 and SIT289 in order of decreasing frequency. Similar to the present study, spoligotype SIT289 was also dominantly isolated in earlier studies conducted in Bahir Dar and its surroundings (24) and Afar Region, Ethiopia (25). Another interesting finding in the present study was, similar to other studies conducted in Ethiopia earlier the ancestor strain SIT523 was found consisting of good numbers of isolates (19, 27). SIT523, characterized by the presence of all 43 spacers in the DR region, was the most frequent pattern, potentially reflecting either ancestral strain persistence or mixed infections. Its persistent prevalence in the population could reflect its ancestral status, suggesting either enhanced biological fitness, successful adaptation to local host populations, or effective human-to-human transmission dynamics. However, another plausible explanation previously suggested in the literature is that the intact spacer pattern observed in SIT523 might represent mixed infections involving multiple strains. Clarifying this point would require molecular tools of higher discriminatory power, such as MIRU-VNTR typing or whole-genome sequencing, which were not employed in our current study. Further molecular epidemiological research is necessary to determine the precise reasons behind the continued high prevalence of SIT523 (30).

Furthermore, SIT149 (T3-ETH) was frequently isolated, consistent with prior reports both in Ethiopia and among Ethiopian immigrants in Denmark (14). This suggests that some strains, such as SIT149, may have a broader geographical distribution, likely due to migration patterns, highlighting the role of population movement in the spread of TB. The persistence of these strains in Ethiopia, even in immigrant populations, further underscores the importance of monitoring and controlling the transmission of *M. tuberculosis* both within and outside the country. Recent studies have shown that *M. tuberculosis* strain distribution in Ethiopia is influenced by a variety of factors, including socio-economic conditions, population migration, and environmental pressures (31, 39).

The *M. tuberculosis* isolated in the present study belonged to four major lineages including the Euro-American, Indo-oceanic, East-African-Indian, and the *M. africanum*. The predominance of the Euro-American lineage (63.64%) among our isolates aligns with findings from previous studies in Ethiopia (18, 19, 27, 28, 39). The predominance of the Euro-American lineage observed in this study aligns with previous reports from different regions of Ethiopia, indicating its widespread distribution across the country. The widespread distribution of this lineage across Ethiopia likely reflects multiple historical and contemporary introduction routes, including European colonization, migration, and trade interactions. While it is tempting to associate specific lineage introductions to historical events such as the Italian occupation (1936–1941), precise attribution is challenging without detailed historical epidemiological data. Therefore, the Euro-American lineage’s broad dissemination in Ethiopia likely results from a combination of factors rather than a single historical event (27). Our study confirmed that one ancestral lineage Indo-Oceanic was circulating in the study population and comprised a relatively high number of strains. Our findings are consistent with the report by Garedew et al. (19) and Baker et al. (41). The presence of the Indo-Oceanic lineage (Lineage 1) in the study population reflects its wide geographical distribution, likely introduced into Ethiopia through historical human migration or trade (19, 41). This supports the idea of regional transmission and evolution of the Indo-Oceanic strain.

The third lineage under which the isolates grouped was East-African-Indian consisting of 14.77% of the isolates. In agreement with the current finding, EAI lineages were reported in a study conducted from farmers in mixed type multipurpose cattle raising region of Ethiopia consisting of 17.7% of the isolates (20). It can also be hypothesized that East-African Indian ancestral strains spread back from Asia to Africa through India as a result of human migration (42). Among the isolates, 1.14% showed a spoligotype pattern consistent with *M. africanum* based on SITVIT2 database matching. However, as no MIRU-VNTR or whole genome sequencing (WGS) was performed, this identification should be interpreted with caution. Our detection of *M. africanum* (1.14%) is notable, as this lineage is rare in East Africa but has been sporadically reported in the west African countries including Guinea-Bissau (43), the Gambia (44), Sierra Leone (45), Senegal (46), Burkina Faso (47), Cameroon (48), Nigeria (49), and Cote D’Ivoire (50). The presence of *M. africanum* could potentially reflect cross-regional transmission events, possibly facilitated by recent human migration or historical trade routes linking West and East Africa. Additionally, zoonotic reservoirs or animal-human interfaces cannot be ruled out, given the complex epidemiology of *Mycobacterium* species. Future studies should explore these possibilities further, employing advanced molecular tools and epidemiological investigations to understand the potential sources and transmission dynamics of *M. africanum* in this region.

Our findings highlight active TB transmission and limited genetic diversity, emphasizing the need for targeted public health interventions. Recommended actions include prioritizing contact tracing, routine molecular surveillance, improving timely diagnosis and treatment adherence, and strengthening worker education. These measures can effectively reduce TB burden and prevent outbreaks among factory workers.

## Conclusion

This study identified a high clustering rate (82.95%) of *Mycobacterium tuberculosis* strains among workers at Metahara Sugar Factory, suggesting active and ongoing TB transmission. All dominant SITs, including SIT523, SIT53, SIT149, and SIT289, belonged to the Euro-American lineage. The relatively limited genetic diversity (31.8%) indicates that a small number of dominant lineages are primarily responsible for local TB cases. The observed limited diversity likely reflects local transmission chains or strain adaptation rather than true absence of diversity, which may be underestimated due to the limitations of spoligotyping. These findings underscore the need for targeted TB prevention and control strategies in factory settings, including rigorous contact tracing, regular molecular surveillance, and enhanced screening and educational interventions. Additionally, employing higher-resolution molecular epidemiological tools such as MIRU-VNTR typing and whole-genome sequencing is critical for accurate tracking of transmission dynamics and effective public health decision-making.

### Limitation of the study

This study has several limitations. First, only smear-positive samples were cultured, potentially missing smear-negative but culture-positive cases and underestimating strain diversity. Second, the use of spoligotyping alone may have overestimated clustering due to its limited discriminatory power. Third, drug susceptibility testing (DST) was not performed, limiting insights into resistance patterns. Fourth, the number of isolates characterized at the molecular level was relatively small. Lastly, previously treated TB cases were excluded, which may have omitted strains related to relapse or reinfection. Future studies should address these gaps using comprehensive culture, DST, and advanced molecular typing methods.

## Data Availability

The datasets generated in this study are available in online repositories, with accession numbers for the spoligotype patterns provided in Tables 3 and 4. Additional raw data supporting the findings are available from the corresponding author upon reasonable request.
